# Environmental and Occupation Factors Associated with Vitamin D Deficiency in Korean Adults: The Korea National Health and Nutrition Examination Survey (KNHANES) 2010–2014

**DOI:** 10.3390/ijerph17249166

**Published:** 2020-12-08

**Authors:** Hye Yin Park, Youn-Hee Lim, Jae Bum Park, Jeongbae Rhie, Soo-Jin Lee

**Affiliations:** 1 Samsung Health Research Institute, Samsung Electronics Co. Ltd., Hwaseong-si 18448, Korea; hyeyinpark@snu.ac.kr; 2 Institute of Environmental Medicine, Seoul National University Medical Research Center, Seoul 03080, Korea; 3 Section of Environmental Health, Department of Public Health, University of Copenhagen, 1014 København, Denmark; younhee.lim@sund.ku.dk; 4 Department of Occupational and Environmental Medicine, Ajou University School of Medicine, Suwon 16499, Korea; jbpark@ajou.ac.kr; 5 Department of Occupational and Environmental Medicine, Dankook University College of Medicine, Cheonan 31116, Korea; rhie76@gmail.com; 6 Department of Occupational and Environmental Medicine, Hanyang University College of Medicine, Seoul 04763, Korea

**Keywords:** environmental exposure, health behavior, occupational exposure, vitamin D

## Abstract

While exposure to sunlight is a well-documented primary source of vitamin D supply, factors leading to vitamin D deficiency vary according to population characteristics. Using nationwide data from the Korea National Health and Nutrition Examination Survey (KNHANES), we aimed to investigate a diverse range of potential factors in association with vitamin D deficiency. Overall, 21,208 participants aged ≥20 years were selected from KNHANES conducted between 2010 and 2014. The associations between various environmental and occupational factors and vitamin D deficiency (defined as serum 25-hydroxyvitamin D [25(OH)D] < 20 ng/mL) were evaluated in logistic regression models after controlling for potential covariates and also after stratification for age and sex. Under given criteria, 15,138 (71.4%) participants were vitamin D deficient. Significant associations were observed between vitamin D deficiency and average environmental temperature and radiation, weekly walking frequency, type of occupation, and shift work. When participants were stratified by age and sex, we observed greater associations of vitamin D deficiency with walking frequency among young males (odds ratio [OR] and 95% confidence interval [95% CI]: 1.24 [1.05–1.47] for those walking <5 times per week compared to those walking ≥5 times per week) and shift work (OR [95% CI]: 1.40 [1.10–1.78] for those working at night compared to those working during the day). We also observed a significant association of vitamin D deficiency with educational attainment (OR [95% CI]: 1.43 [1.09–1.89] for those with ≤middle school compared to those with ≥high school) among older group of females. This study suggests that vitamin D deficiency is related to geographical conditions and subpopulation characteristics. The age and sex-specific associations may urge the effective promotion of vitamin D supply recommendations.

## 1. Introduction

Vitamin D deficiency is a well-documented and common health problem; approximately half the population worldwide is estimated to be vitamin D deficient [1]. Vitamin D deficiency is claimed to affect diverse and complex causes of chronic diseases and conditions, including type 2 diabetes mellitus, cardiovascular diseases, autoimmune disorders, infectious diseases, and cancers [2,3]. In other words, various predictor variables have been repeatedly evaluated and tested for their effects on the aforementioned diseases and conditions, and vitamin D status and supply are no exception [4,5].

While the universal approach to vitamin D supply is the exposure to midday sunlight and its accompanying skin synthesis, supplementation is also recommended for those with reduced sunlight hours during specific times of the year and among susceptible populations [4,6]. Although a broad list, including several environmental and occupational factors, has been proposed [1,2,7], population-specific investigations are still lacking with respect to vitamin D levels. More research on this topic is particularly important because a better understanding of the causes of vitamin D deficiency and their translation to clinical and public health intervention is essential in improving vitamin D status [8,9].

In an attempt to better understand the effects of a diverse range of factors—particularly various individual characteristics and geographical conditions—on vitamin D levels, the current study aimed to identify potential factors as well as any effect modification by age and sex using a nationwide survey.

## 2. Methods

### 2.1. Study Population and Design

The Korea National Health and Nutrition Examination Survey (KNHANES) is a nationally representative survey conducted annually by the Korean Centers for Disease Control and Prevention (KCDC) in South Korea. Details of the data resource profile have been previously described [10,11]. Data from the 5th (2010–2012) and 6th (2013–2015) KNHANES were used in this study. Health interview and examination data were collected annually, and the processed data were publicly available through the KDCD homepage (https://knhanes.cdc.go.kr/knhanes/main.do).

Among 41,102 people who participated in the 2010–2014 surveys, 21,208 subjects aged ≥20 years had available information on serum 25(OH)D levels and were selected for the current analysis. 

### 2.2. Data Collection and Classification

Information on age, sex, lifestyle habits, education level, and occupation, as well as work patterns, was obtained from the health interview data, and anthropometric information and serum 25(OH)D levels were gathered from the health examination data.

Subjects were grouped by age as follows: 20–29, 30–39, 40–49, 50–59, and 60–69 years. Body mass index (BMI) was calculated and treated as a binary variable (< or ≥25 kg/m^2^). Responses to the following questions were reclassified into various categories: previous smoking history (yes or no), weekly walking frequency (< or ≥ 5 times per week), self-rated health (very good/good, average, or bad/very bad), educational attainment (≤ middle school or ≥ high school), current employment status (yes or no), income level (in quartiles), occupation type according to occupation classification code (managers and professional and related workers, clerks, service/sales workers, agricultural/forestry/fishery workers, craft workers/equipment and machine operation and assembly workers, elementary workers, or unemployed (housewife, student, etc.)), shift work (yes or no), and working hours per week (<40, 40–52, or >52 h). 

As the KNHANES provided information on participants’ addresses at the province level (16 provinces in South Korea), meteorological data for the the provinces were obtained from the Korea Meteorological Administration (http://www.kma.go.kr). Average annual levels of meteorological factors for each province, including annual mean temperature, maximum temperature, hours of sunshine, and amount of solar radiation, were obtained. The annual levels of the meteorological factors were then categorized into two levels based on the median values.

According to the Institute of Medicine (IOM) guidelines, a cutoff concentration value of 20 ng/mL (50 nmol/L) was used to define sufficient vitamin D status [12]. Vitamin D deficiency was defined as serum 25(OH)D levels less than 20 ng/mL. 

### 2.3. Statistical Analysis

As KNHANES sampling was conducted in a multi-stage clustered probability design to select representative participants in South Korea, all statistical analyses were performed using sample weights previously constructed by the KCDC. Statistical analyses, including survey procedures, were performed using SAS software version 9.4 (SAS Institute, Inc., Cary, NC, USA) with the statistical significance set at *p* < 0.05. 

Baseline characteristics of study subjects were tested for distribution by serum 25(OH)D levels and vitamin D status. For logistic regression analyses to investigate an association between the different independent variables and vitamin D status, the stepwise selection was performed by selecting covariates at *p* ≤ 0.15 to attain the main-effects model. In the main analysis, effect estimates of odds ratio (OR) with 95% confidence intervals (95% CI) were calculated for all subjects, and in subgroups stratified by sex and age. 

### 2.4. Ethics Statement

This study was conducted in accordance with the Declaration of Helsinki. The KNHANES was approved by the institutional review board at the Korea Center for Disease Control and Prevention (nos. 2010-02CON-21-C; 2011-02CON-06-C; 2012-01EXP-01-2C; 2013-07CON-03-4C; 2013-12EXP-03-5C; and 2015-01-02-6C). All data were collected, and all surveys were conducted with the participants’ written consent. 

## 3. Results

From the 21,208 study subjects, 50.2% (n = 10,640) were 20 to 49 years old, and 55.9% (n = 11,850) were female. Mean serum 25(OH)D levels were lower in females (16.0 ng/mL in females vs. 17.8 ng/mL in males) as well as in the younger age group (16.1 ng/mL in 20–49 years vs. 18.6 ng/mL in those ≥50 years old). Under the given sufficiency criteria of <20 ng/mL, 71.5% of all subjects were vitamin D deficient (76.6% of females and 64.7% of males; 79.8% of those aged 20–49 years; and 62.9% of those 50 years and older) (Table 1).

Among the examined independent variables, significantly lower 25(OH)D levels were observed among individuals with the following characteristics: less frequent walking habits (15.0 ng/mL and 15.9 ng/mL for those who walked < and ≥5 times/week, respectively), higher educational attainment (16.5 ng/mL and 18.5 ng/mL for those with > and ≤ middle school), nighttime shift work (15.9 ng/mL for those with the shift work and 17.3 ng/mL for those with daytime or non-shift work), exposure to lower levels of solar radiation (16.3 ng/mL and 17.7 ng/mL in those exposed to < and ≥ median radiation level, respectively), and lower average environmental temperature (16.5 ng/mL and 17.4 ng/mL for those exposed to < and ≥ median temperature) (Table 1). We observed consistent findings with vitamin D status: A higher proportion of vitamin D deficiency was associated with the educational attainment (76.1% and 61.9% among those with high and low education attainment, respectively). Other categorical variables such as self-rated health, income level, and work hours per week also showed significantly different distributions of 25(OH)D levels, although distributions were not dose-dependent. For example, 25(OH)D levels were lowest in subjects with 40~52 work hours per week (16.7 ng/mL), while higher level was observed at both groups with <40 (17.1 ng/mL) or >52 (17.5 ng/mL) work hours per week (*p*-value for difference in distribution <0.0001).

Through the stepwise model selection, we included the following predictor variables in the main analyses: age, sex, walking frequency, educational attainment, shift work, occupational type, average annual ambient temperature, and annual level of solar radiation. Although BMI was not selected by model fitting, it was included as a priori [4], and sensitivity analyses, excluding BMI, showed consistent results (Supplementary Tables S1 and S2).

ORs on the associations between the potential factors and vitamin D status showed that females were 2.06 times more deficient than males (95% CI: 1.85–2.30), and age was inversely associated with vitamin D deficiency (*p* value for trend <0.0001) (Table 2). In addition, we observed significant associations of vitamin D deficiency with environmental factors such as annual ambient temperature (OR [95% CI]: 1.37 [1.15–1.63] in exposure below the median level) and solar radiation (OR [95% CI]: 1.20 [1.01–1.43] in exposure below the median level) and occupational factors such as shift work (OR [95% CI]: 1.29 [1.12–1.49] in shift workers of any pattern) and indoor work (OR [95% CI]: 2.93 [2.31–3.72] in managers and professional and related workers and 3.09 [2.38–4.00] in clerks and craft workers compared to agricultural, forestry, and fishery workers) (Table 2). 

We observed distinct patterns of the associations between the investigated factors and the status of vitamin D deficiency by age and sex. Some environmental and occupational factors such as annual solar radiation level and occupation type maintained significant correlations across all categorized subgroups. However, associations between vitamin D deficiency and factors such as walking frequency, educational attainment, and shift work were altered when subjects were categorized by age and sex. Statistical significance for those who walked less frequently was only found in males (OR 1.17 [1.03–1.34]) and in the younger subgroup of males (OR 1.24 [1.05–1.47]). Association between lower education level and vitamin D deficiency was observed in only females (OR 1.34 [1.07–1.67]) and in the older subgroup of females (OR 1.43 [1.09–1.89]). In the case of shift work, consistent significance was observed in both the younger and older groups (OR 1.45 [1.20–1.75] for all males; 1.40 [1.10–1.78] in the younger subgroup and 1.46 [1.11–1.92] in the older subgroup) (Table 3).

## 4. Discussion

While vitamin D deficiency is prevalent throughout South Korea, factors affecting vitamin D deficiency showed distinctive patterns of association. When subjects were grouped by age and sex, young adult females showed the highest incidence of vitamin D deficiency, confirming the results of a previous study on vitamin D trends using data from KNHANES 2008–2014 [11]. The association of occupational factors such as shift work and outdoor work was also confirmed, as reported in an earlier study based on data from KNHANES 2010–2012 [13]. The current study also addressed the association of environmental factors such as average temperature and solar radiation level as well as lifestyle factors (e.g., outdoor exercise in terms of walking frequency) and socioeconomic factors (e.g., education level), and observed statistical significance. 

The most noteworthy finding of the current study is that different population subgroups of age and sex showed contrasting factors associated with vitamin D status. When subjects were categorized with respect to these non-modifiable confounders, statistical significance for outdoor physical activity level and education level remained only in the younger male and older female groups, respectively. Association between shift work and vitamin D deficiency remained robust in the male groups but not in the female groups. For geographical and climatic factors, a universal exposure source and association patterns were not distinctively altered by these categorizations. 

While the association results found in the current study are appreciable, caution is needed in interpreting the findings. For example, mean deficient values for vitamin D deficient subjects ranged between 15 and 18.5 ng/mL, and although statistically significant, subtle differences in mean levels may not be of biological importance. On the other side, slightest shift of distribution—i.e., mean vitamin D level—in population basis is of marked change in national level, which is meaningful in scope of public health policy and management. Thus, any tested factors that show statistical significance in the association analyses are important candidates in future causation studies however small the estimated effect.

Unfortunately, not many studies on this topic (other than those on occupational factors) are well-documented. To our knowledge, reports of quantitative evaluation on such dependent variables are rare. While several reports have confirmed the association between physical inactivity and vitamin D deficiency [14,15], a single study on 67 female shift-working nurses revealed that ORs of vitamin D levels for 37 physically active females were not statistically significant (0.97 [0.73–1.29]) compared with those for 26 physically inactive females [16]. Evidence supporting the effect of socioeconomic factors (e.g., education level) on vitamin D status is even more scarce. Nonetheless, the independent variable in the current study could be interpreted as behaviorally or conditionally related with the universal cause of vitamin D deficiency, i.e., reduced skin synthesis [1]. Since current references are insufficient, further research is required to validate the categorized variables of physical activity (e.g., walking frequency) and education level. In the case of walking frequency, while a threshold of walking 5 times per week was selected by statistical methods, investigation on the number of times (i.e., 5 times per week) or the dose–response effect on health has already been carried out [17,18]. Additionally, caution is necessary when interpreting the effect of education level in the current study. While the education level in South Korea is the highest among the Organisation for Economic Co-operation and Development countries [19], statistical significance was observed in both the groups with a maximum education level of “middle school and below” and with an education level of “high school and above.” While low education level is not a common occurrence in the evaluated population, statistical modelling showed more robustness with this categorization. Significance was no longer observed when tested separately with categorization by higher education levels (i.e., “high school and below” and “college and above”) (results not shown).

Employment status or economic activity is a significant component of the modern lifestyle. In comparison with other behavioral/conditional measurement parameters, information regarding employment status can be more easily and precisely collected and classified. As a result, a substantial number of studies investigating the relationship between different occupations and vitamin D deficiency have been updated [7,13,16,20,21]. A large-scale systematic review of 71 peer-reviewed articles showed that the level of vitamin D deficiency was 1.7 and 1.6 times higher in shift workers and indoor workers, respectively, than in outdoor workers [7]. An updated systematic review also stated the consistency of shift workers and indoor workers as an occupationally susceptible group for vitamin D deficiency [21]. Another study that used data from the 2010–2012 KNHANES to investigate occupational factors verified that vitamin D deficiency was prevalent among wage workers, shift workers, and office workers [13]. In agreement with our study, this study showed that a significant relationship was found in male shift workers but not in female shift workers. A more recent cross-sectional study of female-only health care workers showed that shift work had only a small impact on vitamin D levels (OR for daytime work with reference to nighttime shift work: 1.24 [0.91–1.69]), which also supports the current study results [16]. Occupation type was another defining factor in the current study, with significant associations across all age and sex groups. While the aforementioned systematic review reported a specific occupational group (healthcare professionals, including healthcare students and medical residents) to have the highest rate of vitamin D deficiency [7], a separate cross-sectional study that compared general occupation and fisherman groups, with adjustment for residential area in longitudes, showed that serum 25(OH)D levels were 1.7 times higher, and vitamin D insufficiency/deficiency was 20% less in the fisherman group than in the general occupation group [22]. While occupation type is classified into seven separate groups in the current study, it was also confirmed that all other “relatively indoor” occupations were more strongly correlated with vitamin D deficiency than “relatively outdoor” occupations such as agricultural, forestry, and fishery jobs. 

It is also notable that while BMI is suggested as a considerable effect modifier [4], related significant associations were not found in the current study population. One possible explanation is that the prevalence of obesity is relatively low in South Korea compared to the U.S. and Europe where most of the previous evaluations were performed (8.5% in South Korea vs. 40.0% in U.S. in those aged 20–39 years [23,24], by definition of BMI ≥ 30 kg/m^2^). Thus, South-Korea-based studies have a smaller chance of finding any significant relationship with BMI [25]. 

It is also notable that while significantly different distribution of 25(OH)D levels were observed across self-rated health, income level and work hours per week, the distribution patterns were not dose-dependent. While the patterns explain the non-selection of such variables in statistical modeling for the association analysis, further investigation on relationship between such variables and vitamin D status in other populations deserves consideration. 

Moreover, while lifestyle factors such as dietary habits or smoking status are commonly tested for association with vitamin D status, they were eliminated in the statistical modeling. Dietary habits such as fish consumption are related to one of the obvious sources of vitamin D, and future internationally comparable studies addressing these variables may be encouraged. At the same time, dietary intake of vitamin D by natural food is relatively a minor source of vitamin D, with the maximum uptake of 200IU per day [14]. With high proportion of vitamin D deficient subjects in the current study, minimal influence is expected with exclusion of information on dietary habits [26]. As in the case of smoking status, while vitamin D levels were higher in ever-smokers than in never-smokers (17.5 ng/mL vs. 16.5 ng/mL, *p* < 0.0001), they did not meet the statistical criteria for model fitting in the association analysis. The statistical significance of smoking in the univariate model and then the non-significance in the multivariate model could likely be due to confounding by sex and others. Altogether, continued research on relationship between various lifestyle factors and vitamin D status is still highly recommended. 

Strengths of this study include a nationally representative sample, a large sample size accumulated for over a five-year period, and standardized and consistent outcome measurements. Additionally, this is one of the few studies that attempted to investigate as many potential factors of vitamin D deficiency as possible. Cumulatively, findings of this study maximized the utilization of available data.

Several limitations should be considered when interpreting the results of the current study. First, because of the cross-sectional design, causality between the statistically significant factors and vitamin D status is not guaranteed. Second, as subject information on dates and addresses were unavailable, only provincial- and annual-level meteorological data could be utilized; caution is needed when comprehending the effects of solar radiation and average environmental temperature on an individual’s vitamin D status. Moreover, while seasonality is an important confounder for assessing vitamin D status [4], information regarding what time of the year the blood samples were drawn was unavailable to the public and could not be investigated in the current study. Due to the blinded information on the season of blood sample collection, the study results may not be exempt from criticism that the statistical differences according to different characteristics could represent real biological significance. On the other hand, an internal report published elsewhere showed that sample drawings were evenly distributed throughout the year [11], indicating some uniform distribution of seasonality regardless of the lack of information on individuals’ sample draw date. Still, even distribution of 25(OH)D sampling is not guaranteed despite the well-documented sampling process for selecting subjects testing for vitamin D levels [10,11]. From 41,102 KNHANES participants, 31,319 participants were 20 years and over, and 24,532 participants had vitamin D levels available; a subset of 21,208 subjects were both over 20 years and had 25(OH)D levels available. When subjects with and without 25(OH)D levels were compared in all age groups and in groups confined to 20 years and over, most of the independent variables tested for the association analyses showed statistically significant *p*-values at <0.05 (results not shown). Finally, as this survey represents a single-race population within a relatively small geographical area, careful consideration is needed when generalizing the study results. 

## 5. Conclusions

In conclusion, while universal demand for vitamin D supply is evident in the modern world, it is important to consider probable factors associated with vitamin D status among different population subgroups. Clinical interventions regarding vitamin D status may be more effective if recommendations are targeted toward specific groups (e.g., developing and delivering educational resources to groups of young women or promoting outdoor activity in male groups of any age). 

## Figures and Tables

**Table 1:** Baseline characteristics of study subjects classified (1) by serum 25(OH)D levels and (2) vitamin D status.

	N	(1) Serum 25(OH)D		(2) Vitamin D Status				
		Mean ± SE (ng/mL)	*p* Value	Non-Deficient (≥20 ng/mL)		Deficient (<20 ng/mL)		*p* Value
				n	%	n	%	
All subjects	21,208	17.1 ± 0.1		6070	28.6	15,138	71.4	
Age group								
20~40s	10,640	16.1 ± 0.1	<0.0001	2150	20.2	8490	79.8	<0.0001
≥50s	10,568	18.6 ± 0.1		3920	37.1	6648	62.9	
Sex								
Male	9358	17.8 ± 0.1	<0.0001	3302	35.3	6056	64.7	0.0001
female	11,850	16.0 ± 0.1		2768	23.4	9082	76.6	
Body mass index								
≥25 kg/m^2^	14,361	17.1 ± 0.1	0.161	4088	28.5	10,273	71.5	0.3061
<25 kg/m^2^	6777	16.9 ± 0.1		1969	29.1	4808	70.9	
Ever smoking								
No	11,995	16.5 ± 0.1	<0.0001	3024	25.2	8971	74.8	<0.0001
Yes	8529	17.5 ± 0.1		2864	33.6	5665	66.4	
Walking frequency								
≥5 times/week	9219	15.9 ± 0.2	0.006	2739	29.7	6480	70.3	0.0669
<5 times/week	11,223	15.5 ± 0.3		3135	27.9	8088	72.1	
Self-rated health								
“Very good”, “Good”	6761	17.2 ± 0.1	0.005	1951	28.9	4810	71.1	0.0736
“Average”	9913	16.8 ± 0.1		2758	27.8	7155	72.2	
“Bad”, “Very bad”	3786	17.0 ± 0.2		1171	30.9	2615	69.1	
Education level								
≤Middle school graduate	6956	18.5 ± 0.2	<0.0001	2650	38.1	4306	61.9	<0.0001
≥High school	13,473	16.5 ± 0.1		3220	23.9	10,253	76.1	
Economic activity status								
Yes (employed, etc.)	12,480	17.2 ± 0.1	<0.0001	3737	29.9	8743	70.1	<0.0001
No (unemployed, etc.)	7952	16.5 ± 0.1		2133	26.8	5819	73.2	
Income level								
1st(lowest) quartile	5138	17.0 ± 0.2	0.0029	1559	30.3	3579	69.7	0.0201
2nd quartile	5284	16.8 ± 0.1		1473	27.9	3811	72.1	
3rd quartile	5307	16.8 ± 0.1		1428	26.9	3879	73.1	
4th(highest) quartile	5247	17.3 ± 0.1		1556	29.7	3691	70.3	
Occupation type								
Managers, professional, and related workers	2717	16.3 ± 0.2	<0.0001	591	21.8	2126	78.2	<0.0001
Clerks	1803	16.0 ± 0.2		380	21.1	1423	78.9	
Service/sales workers	2568	16.5 ± 0.2		614	23.9	1954	76.1	
Agricultural, forestry, and fishery workers	1558	21.6 ± 0.4		906	58.2	652	41.8	
Craft workers, equipment and machine operation and assembling workers	2017	18.0 ± 0.2		658	32.6	1359	67.4	
Elementary workers	1767	17.4 ± 0.2		569	32.2	1198	67.8	
Unemployed (housewife, student, etc.)	7952	16.6 ± 0.1		2133	26.8	5819	73.2	
Shiftwork								
No	11,620	17.3 ± 0.1	<0.0001	3513	30.2	8107	69.8	<0.0001
Yes	2365	15.9 ± 0.2		519	21.9	1846	78.1	
Work hours per week								
<40 h	4699	17.1 ± 0.1	<0.0001	1451	30.9	3248	69.1	<0.0001
40~52 h	5638	16.7 ± 0.1		1439	25.5	4199	74.5	
>52 h	3727	17.5 ± 0.2		1165	31.3	2562	68.7	
Avg. solar radiation (med: 10.01MJ/m^2^)								
≥1.01MJ/m^2^	10,444	17.7 ± 0.2	<0.0001	3417	32.7	7027	67.3	<0.0001
<1.01MJ/m^2^	10,764	16.3 ± 0.1		2653	24.6	8111	75.4	
Avg. temperature (med: 120.3 °C)								
≥12.3 °C	10,192	17.4 ± 0.2	0.0002	3147	30.9	7045	69.1	0.0139
<12.3 °C	11,016	16.5 ± 0.2		2923	26.5	8093	73.5	
Max. temperature (med: 350.4 °C)								
≥35.4 °C	10,304	17.0 ± 0.2	0.6295	2880	28	7424	72	0.7326
<35.4 °C	10,904	16.9 ± 0.2		3190	29.3	7714	70.7	
Avg. sunshine amount (med: 460.4%)								
≥46.4%	8033	16.7 ± 0.2	0.095	2000	24.9	6033	75.1	<0.0001
<46.4%	13,175	17.2 ± 0.2		4070	30.9	9105	69.1	

**Table 2:** Logistic regression analysis of association between occupational and environmental factors and vitamin D deficiency (<20 ng/mL) from KNHANES 2010-2014.

Characteristic	Category	OR (95% CI)
Sex (Reference Male)	Female	2.06 (1.85–2.30)
Age (Reference ≥70 years)	20–29 years	2.72 (2.04–3.62)
	30–39 years	1.80 (1.38–2.35)
	40–49 years	1.41 (1.09–1.82)
	50–59 years	1.03 (0.82–1.30)
	60–69 years	0.96 (0.78–1.19)
Body mass index (Reference <25 kg/m^2^)	≥25 kg/m^2^	1.04 (0.94–1.16)
Walking frequency (Reference ≥5 times/week)	<5 times/week	1.14 (1.03–1.27)
Education level (Reference ≥High school)	≤Middle school	1.13 (0.97–1.32)
Shiftwork (Reference No)	Yes	1.29 (1.12–1.49)
Occupation type (Reference Agricultural, forestry and fishery workers)	Managers, professional and related workers	2.93 (2.31–3.72)
	Clerks	3.09 (2.38–4.00)
	Service/sales workers	2.60 (2.07–3.28)
	Craft workers, equipment and machine operation and assembling workers	2.47 (1.94–3.14)
	Elementary workers	2.10 (1.67–2.65)
	Unemployed (housewife, student, etc.)	2.99 (2.32–3.86)
Annual average temperature (Reference ≥Median)	<Median (1.01MJ/m^2^)	1.20 (1.01–1.43)
Annual solar radiation (Reference ≥Median)	<Median (12.3 °C)	1.37 (1.15–1.63)

**Table 3:** Logistic regression analysis of association between occupational and environmental factors and vitamin D deficiency (<20 ng/mL) from KNHANES 2010–2014, stratified by gender and age.

	(1) Stratified by Gender		(2) Stratified by Gender and Age Group			
	Males (n = 10,598)	Females (n = 15,671)	Male, 20–40s (n = 4621)	Female, 20–40s (n = 7280)	Male, 50s+ (n = 5977)	Female, 50+ (n = 8391)
Age (Reference ≥70 years)						
20–29 years	2.25 (1.62–3.13)	3.39 (2.15–5.33)				
30–39 years	1.54 (1.13–2.10)	2.12 (1.45–3.10)				
40–49 years	1.14 (0.85–1.54)	1.83 (1.28–2.62)				
50–59 years	0.88 (0.67–1.15)	1.24 (0.91–1.68)				
60–69 years	0.87 (0.67–1.13)	1.03 (0.77–1.39)				
Body mass index (Reference <25 kg/m^2^)						
≥25 kg/m^2^	1.03 (0.91–1.18)	1.09 (0.92–1.29)	1.04 (0.87–1.24)	1.03 (0.78–1.35)	1.003 (0.82–1.22)	1.17 (0.95–1.44)
Walking frequency (Reference ≥5 times/week)						
<5 times/week	1.17 (1.03–1.34)	1.09 (0.93–1.28)	1.24 (1.05–1.47)	1.25 (0.99–1.57)	1.04 (0.85–1.27)	0.95 (0.77–1.16)
Education level (Reference ≥High school)						
≤Middle school	1.03 (0.85–1.26)	1.34 (1.07–1.67)	0.99 (0.68–1.42)	1.25 (0.84–1.85)	1.10 (0.87–1.37)	1.43 (1.09–1.89)
Shiftwork (Reference No)						
Yes	1.45 (1.20–1.75)	1.03 (0.83–1.28)	1.40 (1.10–1.78)	1.19 (0.89–1.60)	1.46 (1.11–1.92)	0.80 (0.58–1.10)
Occupation type (Reference Agricultural, forestry and fishery workers)						
Managers, professional and related workers	3.31 (2.46–4.46)	2.33 (1.67–3.24)	3.47 (2.12–5.65)	2.67 (1.53–4.66)	2.78 (1.93–4.02)	1.50 (0.89–2.55)
Clerks	3.40 (2.48–4.68)	2.59 (1.80–3.71)	3.55 (2.12–5.96)	2.98 (1.68–5.27)	2.55 (1.66–3.90)	2.03 (1.05–3.90)
Service/sales workers	2.92 (2.15–3.96)	2.21 (1.67–2.94)	2.91 (1.75–4.86)	2.51 (1.44–4.38)	2.69 (1.87–3.86)	2.06 (1.50–2.83)
Craft workers, equipment and machine operation and assembling workers	2.63 (1.97–3.50)	2.48 (1.67–3.68)	2.57 (1.59–4.16)	2.94 (1.48–5.83)	2.69 (1.95–3.73)	2.23 (1.33–3.74)
Elementary workers	2.07 (1.51–2.85)	2.03 (1.55–2.66)	1.76 (1.02–3.03)	1.85 (1.04–3.26)	2.43 (1.71–3.46)	2.23 (1.65–3.02)
Unemployed (housewife, student, etc.)	3.61 (2.57–5.07)	2.27 (1.66–3.12)	3.41 (1.95–5.95)	2.50 (1.41–4.43)	3.87 (2.61–5.72)	2.20 (1.47–3.28)
Annual average temperature (Reference ≥Median)						
<Median (1.01MJ/m^2^)	1.17 (0.97–1.42)	1.26 (1.002–1.59)	1.14 (0.91–1.44)	1.27 (0.95–1.71)	1.23 (0.96–1.56)	1.28 (0.96–1.69)
**Annual solar radiation (Reference ≥Median)**						
<Median (12.3 ℃)	1.40 (1.15–1.70)	1.31 (1.04–1.64)	1.36 (1.08–1.72)	1.28 (0.95–1.71)	1.48 (1.16–1.89)	1.37 (1.03–1.81)

## Supplementary Materials

The following are available online at https://www.mdpi.com/1660-4601/17/24/9166/s1, Table S1: Logistic regression analysis of association between occupational and environmental factors (excluding body mass index) and vitamin D deficiency (<20 ng/mL) from KNHANES 2010–2014. Table S2: Logistic regression analysis of association btw occupational & environmental factors (excluding body mass index) and vitamin D deficiency (<20 ng/mL) from KNHANES 2010–2014, stratified by gender and age.
