# Supplementary material for: Environmental and Occupation Factors Associated with Vitamin D Deficiency in Korean Adults: The Korea National Health and Nutrition Examination Survey (KNHANES) 2010–2014
Source: Int J Environ Res Public Health. 2020 Dec 8;17(24):9166. doi: 10.3390/ijerph17249166 (PMC7762981; doi:10.3390/ijerph17249166)
Supplement: Supplementary file 1 [file ijerph-17-09166-s001.pdf]

Table S1. Logistic regression analysis of association between occupational and environmental factors (excluding body mass index) and vitamin D deficiency (<20ng/ml) from KNHANES 2010–2014.

		**OR (95% CI)**
Sex (ref: Male)	Female	2.05 (1.84–2.28)
Age (ref: ≥70 years)	20–29 years	2.71 (2.03–3.61)
	30–39 years	1.81 (1.39–2.36)
	40–49 years	1.40 (1.09–1.81)
	50–59 years	1.03 (0.82–1.30)
	60–69 years	0.97 (0.78–1.20)
Walking frequency (ref: ≥5 times/week)	<5 times/week	1.15 (1.04–1.27)
Education level (ref: ≥High school)	≤Middle school	1.14 (0.98–1.32)
Shiftwork (ref: No)	Yes	1.30 (1.12–1.50)
Occupation (ref: Agricultural, forestry and fishery workers)	Managers, professional and related workers	2.94 (2.32–3.72)
	Clerks	3.09 (2.38–4.00)
	Service/sales workers	2.60 (2.07–3.28)
	Craft workers, equipment and machine operation and assembling workers	2.47 (1.94–3.14)
	Elementary workers	2.10 (1.66–2.64)
	Unemployed(housewife, student, etc.)	2.98 (2.31–3.84)
Average temperature (ref: ≥Median)	<Median (12.3 °C)	1.20 (1.01–1.43)
Average radiation (ref: ≥Median)	<Median (1.01MJ/m^2)	1.37 (1.14–1.63)

Table S2. Logistic regression analysis of association btw occupational & environmental factors (excluding body mass index) and vitamin D deficiency (<20 ng/ml) from KNHANES 2010–2014, stratified by gender and age.

	**(1) Stratified by Gender**		**(2) Stratified by Gender and Age Group**			
	**Males (n = 10,598)**	**Females (n = 15,671)**	**Male, 20–40s (n = 4621)**	**Female, 20–40s (n = 7280)**	**Male, 50s+ (n = 5977)**	**Female, 50+ (n = 8391)**
Age (ref: ≥70 years)						
20–29 years	2.26 (1.62–3.14)	3.32 (2.11–5.23)				
30–39 years	1.56 (1.14–2.12)	2.12 (1.45–3.11)				
40–49 years	1.15 (0.85–1.54)	1.82 (1.27–2.60)				
50–59 years	0.88 (0.67–1.15)	1.24 (0.91–1.68)				
60–69 years	0.87 (0.67–1.14)	1.04 (0.78–1.39)				
Walking frequency (ref: ≥5 times/week)						
<5 times/week	1.18 (1.03–1.34)	1.09 (0.93–1.28)	1.25 (1.05–1.47)	1.25 (0.99–1.58)	1.05 (0.86–1.28)	0.95 (0.77–1.16)
Education level (ref: ≥High school)						
≤Middle school	1.03 (0.85–1.26)	1.34 (1.07–1.67)	0.99 (0.69–1.43)	1.29 (0.87–1.91)	1.09 (0.87–1.37)	1.39 (1.05–1.83)
Shiftwork (ref: no)						
Yes	1.45 (1.20–1.75)	1.03 (0.83–1.28)	1.40 (1.10–1.78)	1.20 (0.89–1.60)	1.46 (1.11–1.92)	0.8 (0.59–1.10)
Occupation (ref: Agricultural, forestry and fishery workers)						
Managers, professional and related workers	3.32 (2.47–4.46)	2.32 (1.67–3.23)	3.46 (2.12–5.65)	2.64 (1.52–4.61)	2.79 (1.94–4.02)	1.53 (0.91–2.58)
Clerks	3.41 (2.48–4.68)	2.58 (1.79–3.71)	3.55 (2.12–5.96)	2.93 (1.66–5.20)	2.56 (1.67–3.91)	2.04 (1.06–3.92)
Service/sales workers	2.93 (2.16–3.97)	2.20 (1.66–2.92)	2.91 (1.74–4.85)	2.48 (1.42–4.34)	2.71 (1.89–3.87)	2.05 (1.49–2.82)
Craft workers, equipment and machine operation and assembling workers	2.63 (1.98–3.51)	2.48 (1.67–3.69)	2.57 (1.59–4.17)	2.94 (1.48–5.82)	2.70 (1.95–3.73)	2.24 (1.34–3.76)
Elementary workers	2.08 (1.52–2.86)	2.01 (1.53–2.63)	1.76 (1.03–3.03)	1.78 (1.01–3.15)	2.43 (1.71–3.47)	2.22 (1.64–3.00)
Unemployed(housewife, student, etc.)	3.59 (2.56–5.03)	2.26 (1.65–3.10)	3.39 (1.94–5.92)	2.46 (1.38–4.37)	3.81 (2.57–5.63)	2.19 (1.47–3.26)
Average temperature (ref: ≥Median)						
<Median (12.3℃)	1.18 (0.97–1.42)	1.26 (0.999–1.59)	1.15 (0.91–1.44)	1.27 (0.94–1.70)	1.22 (0.96–1.56)	1.28 (0.97–1.70)
Average radiation (ref: ≥Median)						
<Median (1.01MJ/m²)	1.40 (1.15–1.70)	1.29 (1.03–1.62)	1.37 (1.08–1.73)	1.26 (0.94–1.70)	1.48 (1.16–1.88)	1.35 (1.02–1.79)
